# Dual Inhibition of Phosphodiesterase 3 and 4 Enzymes by Ensifentrine Protects against MRSA-Induced Lung Endothelial and Epithelial Dysfunction

**DOI:** 10.3390/cells13211750

**Published:** 2024-10-23

**Authors:** Mohammed Yaman Al Matni, Lucille Meliton, Steven M. Dudek, Eleftheria Letsiou

**Affiliations:** Division of Pulmonary, Critical Care, Sleep and Allergy, University of Illinois Chicago, Chicago, IL 60612, USA; malmat2@uic.edu (M.Y.A.M.); lmeliton@uic.edu (L.M.); sdudek@uic.edu (S.M.D.)

**Keywords:** ARDS, acute lung injury, ensifentrine, RPL554, phosphodiesterase, permeability, inflammation, alveolar epithelium, endothelial

## Abstract

Acute Respiratory Distress Syndrome (ARDS) is a severe lung condition with a high mortality rate for which there are no effective therapeutics. The failure of the alveolar–capillary barrier, composed of lung endothelial (EC) and alveolar epithelial (AEC) cells, is a critical factor leading to excessive inflammation and edema characteristic of acute lung injury (ALI) pathophysiology. Phosphodiesterases (PDE) are enzymes well-recognized for their roles in regulating endothelial permeability and inflammation. Although PDE inhibitors are used as therapeutics for inflammatory diseases like COPD (chronic obstructive pulmonary disease), their efficacy in treating ARDS has not yet been established. In this study, we investigated the effects of ensifentrine, an FDA-approved novel dual PDE 3/4 inhibitor, on lung endothelial and epithelial dysfunction caused by methicillin-resistant *S. aureus* (MRSA), a pathogen involved in bacterial ARDS. Human primary lung endothelial cells and alveolar epithelial cell lines (A549 and immortalized AEC) were treated with heat-killed MRSA, and their responses were assessed in the presence or absence of ensifentrine. Ensifentrine given either pre- or post-exposure attenuated MRSA-induced increased lung endothelial permeability. VE-cadherin junctions, which serve to stabilize the EC barrier, were disrupted by MRSA; however, ensifentrine effectively prevented this disruption. Pre-treatment with ensifentrine protected against MRSA-induced EC pro-inflammatory signaling by inhibiting the expression of VCAM-1, ICAM-1, and by reducing the IL-6 and IL-8 release. In AEC, MRSA caused the upregulation of ICAM-1, the activation of NF-kB, and the production of IL-8, all of which were inhibited by ensifentrine. These results indicate that the dual inhibition of phosphodiesterases 3 and 4 by ensifentrine is barrier protective and attenuates MRSA-induced inflammation in both lung endothelial and epithelial cells. The PDE3/4 inhibitor ensifentrine may represent a promising novel strategy for the treatment of MRSA-induced ARDS.

## 1. Introduction

Acute respiratory distress syndrome (ARDS) is a condition marked by damage to the lung’s alveolar–capillary barrier, leading to the flooding of alveoli with protein-rich fluid and resulting in respiratory failure [1]. ARDS comprises roughly 10% of all ICU admissions and continues to have a significant mortality rate of approximately 40%. Despite decades of research, there is still no effective approved medication for treating ARDS, and supportive care remains the primary management approach [2,3]. Although a wide range of conditions can result in ARDS, including acid aspiration, severe trauma, and repeated blood transfusions, sepsis and pneumonia are the main risk factors for the syndrome, with *Streptococcus pneumoniae* and *Staphylococcus aureus* being the most common bacteria causing pneumonia-induced ARDS [1,4,5]. Alveolar epithelial cells (AEC), along with lung endothelial cells (EC), form the lung’s alveolar–capillary barrier, and are primary targets for bacteria and other respiratory pathogens. In acute lung injury (ALI), a series of events that include AEC and EC activation and the production of pro-inflammatory mediators lead to lung barrier disruption and excessive immune cell recruitment [1]. Strategies that inhibit these processes by preserving AEC and EC function following injury represent promising therapeutics for ARDS.

Phosphodiesterases (PDEs) are a family of enzymes that catalyze the hydrolysis of cyclic adenosine monophosphate (cAMP) and cyclic guanosine monophosphate (cGMP), which are important intracellular second messengers regulating several signaling pathways and cellular functions [6]. The PDE family consists of 11 groups, for which numerous specific inhibitors (PDEis) have been developed. Among these, PDE 3 and 4 inhibitors have attracted significant interest due to their cytoprotective and anti-inflammatory actions [7,8,9,10]. Several FDA-approved PDE3 or 4 inhibitors are clinically available to treat various conditions including chronic obstructive pulmonary disease (COPD), congestive heart failure, and peripheral artery disease [11,12,13]. In ARDS, there has been particular interest in PDEs and how their modulation can impact the underlying pathogenetic mechanisms [14]. This is due to extensive research demonstrating that the inhibition of PDE 3 or 4 suppresses the increased endothelial permeability, epithelial dysfunction, inflammatory mediator release, and immune cell recruitment that characterize ALI pathophysiology [14,15,16,17]. Despite these promising effects, there has been no significant progress in assessing the efficacy of PDE 3 or 4 inhibitors in treating ARDS [14].

Ensifentrine (RPL554) is a novel PDE inhibitor that was recently approved in June 2024 as a maintenance treatment for COPD (administered via a nebulizer) [18,19]. Ensifentrine has a novel mechanism of action by inhibiting both PDE 3 and 4, resulting in combined bronchodilator and anti-inflammatory actions [20,21]. Compared to other FDA-approved PDE inhibitors that selectively target either PDE3 or PDE4 and exhibit limited tolerability, ensifentrine demonstrates an exemplary clinical profile in terms of tolerability, safety, and efficacy [22]. This makes it a highly promising therapeutic candidate for multiple indications, including ARDS. Our prior work has demonstrated that methicillin-resistant *Staph aureus* (MRSA), an ARDS-causing pathogen, is a potent inflammatory stimulus causing lung endothelial barrier disruption and inflammation [23,24]. Whether the dual inhibition of PDE3 and 4 by ensifentrine can ameliorate MRSA’s injurious effects in the lung is unknown. Therefore, in the present study, we aimed to investigate the effects of ensifentrine on lung endothelial and alveolar epithelial dysfunction caused by MRSA to explore the potential efficacy of this intervention in models of ARDS.

## 2. Materials and Methods

Cell culture and treatments. Human pulmonary artery endothelial cells (HPAEC, Cat#CC2539) and human lung microvascular endothelial cells (HLMVEC, Cat#CC2527) were obtained from Lonza (Walkersville, MD, USA) and cultured in EBM-2 Basal Medium supplemented with EGM-2 SingleQuots supplements (Lonza) and 10% fetal bovine serum (FBS) (MilliporeSigma, St Louis, MO, USA). A549 were grown in RPMI (with L-glutamine) (Corning, Corning, NY, USA) supplemented with 5% FBS. Immortalized alveolar epithelial cells (iAEC, Cat# H-6053IM) and corresponding media (complete human epithelial cell medium, Cat# H6621) were purchased from CellBiologics (Chicago, IL, USA). All of the cells were grown at 37 °C in a 5% CO_2_ incubator. Endothelial cells were used for experiments at passages 5–7. Before treatments, EC and epithelial cells (A549, iAEC) were incubated in 2% FBS media and FBS-free media, respectively, for 2 h. A 10 mM stock of ensifentrine (Cat#HY-119708, MedChemExpress, Monmouth Junction, NJ, USA) was made in DMSO, and aliquots were kept in −80 °C for up to 1 month. Cells were treated with ensifentrine or DMSO for 1 h (unless noted) followed by heat-killed MRSA treatment (2.5 × 10^8^ CFU/mL). In separate experiments, cells were pre-treated with ESI-08, an EPAC (Exchange Protein Activated by Cyclic AMP) antagonist (Cat#HY-136172, MedChemExpress) [25], prior to ensifentrine and MRSA. The DMSO concentration for all experiments was less than 0.5%. The USA300 CA-MRSA wild-type (LAC) strain used in this study was kindly provided by Dr. Jiwang Chen (UIC). Heat-killed bacteria (HK-MRSA) were prepared as described previously [23,24]. The HK-MRSA was diluted in PBS, aliquoted, and stored at −80 °C until the day of the experiment.

Electric Cell–substrate Impedance Sensing (ECIS). EC monolayer barrier integrity was assessed using the ECIS assay (Applied Biophysics, Troy, NY, USA), as we have described previously [23]. Briefly, EC were seeded into 8-well ECIS arrays and grown to confluency before indicated treatments. Transendothelial electrical resistance (TER) values were measured over time. For data analysis, normalized TER was plotted versus time. To quantify the changes, the area under the curve (AUC) was calculated for each condition for the time period of 2–20 h, or as indicated. For each independent experiment, the AUC values of the treated conditions were normalized to the control.

XPerT permeability assay. The gap formation in the endothelial monolayers was assessed using the XPerT permeability assay as described [24,26,27], with some modifications. Briefly, confluent HPAEC grown on biotinylated gelatin coated 12-well plates were pre-treated with ensifentrine (5 μM) or DMSO for 1 h, followed by HK-MRSA treatment (2.5 × 10^8^ CFU/mL). Then, 20 h later, FITC-conjugated avidin (Thermo Fisher Scientific, Skokie, IL, USA) was added to the media (7.5 μg/mL) for 2 min. The cells were washed quickly in pre-warmed 3.7% paraformaldehyde (PFA) (Boston BioProducts, Milford, MA, USA), and then fixed in 3.7% PFA for 15 min. Images were taken with a REVOLVE microscope (Discover Echo Inc, San Diego, CA, USA) with a 20× Olympus objective. Gap formation was defined by green fluorescence signal (matrix bound FITC-avidin), and Image J, v1.53k (National Institutes of Health, Bethesda, MD, USA) was used to quantify the area of green immunofluorescence as we have described previously [27].

Western blotting. Following the indicated treatments, the cells were washed in ice-cold PBS and then lysed with RIPA buffer (MilliporeSigma, St Louis, MO, USA) containing protease and phosphatase inhibitors (MilliporeSigma). Protein lysates collected after high-speed centrifugation were mixed with 6× SDS-sample buffer (Boston BioProducts) and boiled for 5 min. Protein samples were loaded into 12–20% SurePAGE gels (GenScript, Piscataway, NJ, USA) and transferred onto PVDF membranes (MilliporeSigma). The BLUEstain Protein Ladder (11–245 kDa) from GoldBio (St. Louis, MO, USA) was used to identify the molecular weights. Membranes were then immunoblotted with primary antibodies ICAM-1 (Cat#A5597, ABclonal, Woburn, MA, USA), VCAM-1 (Cat#sc-8304, Santa Cruz Biotech, Dallas, TX, USA), and phospho-NFkB (Cat#3033, Cell signaling, Danvers, MA, USA) at 4 °C (overnight) followed by secondary anti-rabbit antibody conjugated to HRP from Cell Signaling (room temperature, 1 h). Membranes were then incubated with HRP-conjugated β-actin monoclonal antibody (Cat#HRP-60008, Proteintech, Rosemont, IL, USA) as the loading control. For membrane stripping, we used the Restore PLUS Western blot stripping buffer (Cat#46430, Thermo Fisher) according to the manufacturer’s instructions. Protein expression was detected with Pierce ECL Western blotting substrate (Cat#32106, Thermo Fisher) on HyBlot CL film (Thomas Scientific, Swedesboro, NJ, USA). The blots were analyzed using ImageJ software (v1.53k).

Immunofluorescence. HPAEC were grown on 8-well glass slides (Millicell EZ slide; MilliporeSigma) until confluency. Following the indicated treatments, cells were fixed in 3.7% PFA (Boston BioProducts) for 15 min at room temperature. After washing in PBS, cells were permeabilized in 0.1% triton-X in PBS for 4 min followed by blocking in 3% BSA/PBS for 1 h. Then, cells were incubated for 10 min in room temperature with a Fc receptor blocking solution (Trustain FcX, Biolegend, San Diego, CA, USA) to block non-specific binding from MRSA [28]. Trustain was diluted in 1% BSA/PBS. Primary antibody (VE-cadherin F8; Cat# sc-9989, Santa Cruz Biotech) was then added to the cells (without the removal of the Fc receptor blocking solution) at 1:150 at 4 °C overnight. After washing in PBS, cells were incubated with anti-mouse AlexaFluor-488 secondary antibody from Thermo Fisher Scientific (1:500, 4 °C, 1 h in the dark). After washing, cells were mounted with ProLong DAPI (Thermo Fisher). Imaging was performed using the REVOLVE microscope and a 20× Olympus objective.

Cell viability. Lactate dehydrogenase (LDH) activity was measured in cell supernatants using the Roche Cytotoxicity detection kit (Cat# 11644793001, Millipore Sigma). After collection, cell supernatants were centrifuged at 2000× *g* for 10 min and immediately analyzed for LDH. Absorbance was measured in the microplate reader Spectramax M2e (Molecular Devices, San Jose, CA, USA) at 490 nm with a reference wavelength of 600 nm for 20 min. ΔOD was calculated for each condition.

ELISA. Human IL-6 and IL-8 levels were measured in cell supernatants using ELISA MAX Deluxe set kits from Biolegend (San Diego, CA, USA), according to the manufacturer’s instructions.

Data analysis and statistics. Experiments were performed at least 3 times independently of one another, and the results are expressed as mean ± standard deviation (SD). Data graphing and statistics were conducted using GraphPad Prism software (version 10). Comparisons between groups were made using a *t*-test or one-way analysis of variance (ANOVA) followed by Tukey’s post hoc test. *p* values < 0.05 were considered statistically significant.

## 3. Results

### 3.1. Ensifentrine Enhances the Lung Endothelial Barrier and Protects against MRSA-Induced EC Barrier Disruption

**Figure 1:**
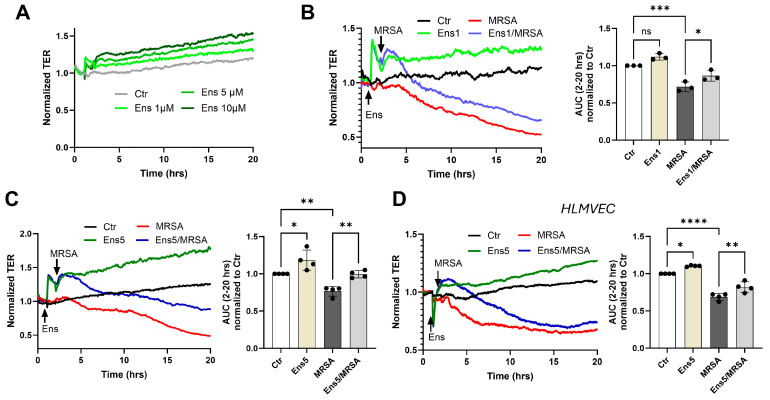
Ensifentrine exhibits barrier-enhancing and barrier-protective properties against MRSA. Human pulmonary artery endothelial cells (HPAEC) (**A**–**C**) or human lung microvascular endothelial cells (HLMVEC) (**D**) were pre-treated with ensifentrine or vehicle (DMSO) for 1 h prior to HK-MRSA challenge (2.5 × 10^8^/mL). The EC barrier was assessed with the ECIS assay. (**A**) Representative TER tracings over time of HPAEC treated with various doses of ensifentrine (1–10 μM) in control cells. (**B**,**C**) Representative TER tracings over time of HPAEC pre-treated with 1 and 5 μM ensifentrine prior to HK-MRSA. The area under the curve was calculated for each condition and normalized to untreated cells (Ctr). (**D**) Representative TER tracings over time of HLMVEC pre-treated with 5 μM ensifentrine prior to MRSA and corresponding quantification. N = 3–4 independent experiments. Data were analyzed using one-way ANOVA, ns not significant, * *p* < 0.05, ** *p* < 0.01, *** *p* < 0.001. **** *p* < 0.0001.

Earlier studies have shown that PDE inhibition can modulate endothelial barrier properties [15]. Here, we examined whether the dual inhibition of PDE3 and 4 by ensifentrine can impact barrier integrity. As shown in Figure 1A, ensifentrine caused a dose-dependent sustained increase in the baseline resistance of HPAEC, which suggests that ensifentrine has potent barrier-enhancement properties. The changes induced by ensifentrine on the barrier were immediate, as illustrated in Supplementary Figure S1. As we have previously shown, MRSA treatment causes a significant decline in TER values over time, indicating an increased EC permeability [23,24]. Ensifentrine pre-treatment at low doses (1 μΜ) partially protected against MRSA-induced barrier disruption (Figure 1B). The effects of ensifentrine were more potent at higher doses of 5 μΜ (Figure 1C) and 10 μM (Supplementary Figure S2A). Both doses potently inhibited MRSA-induced TER reduction over time. For the remaining experiments, a dosage of ≤5 μΜ of ensifentrine was selected, since 10 μΜ induced a small but significant increase in IL-6 levels at baseline (Supplementary Figure S2D), as described below. These data obtained using HPAEC were then confirmed using human lung microvascular EC as depicted in Figure 1D, which shows that ensifentrine exhibits both barrier-enhancing effects at baseline and after MRSA.

**Figure 2:**
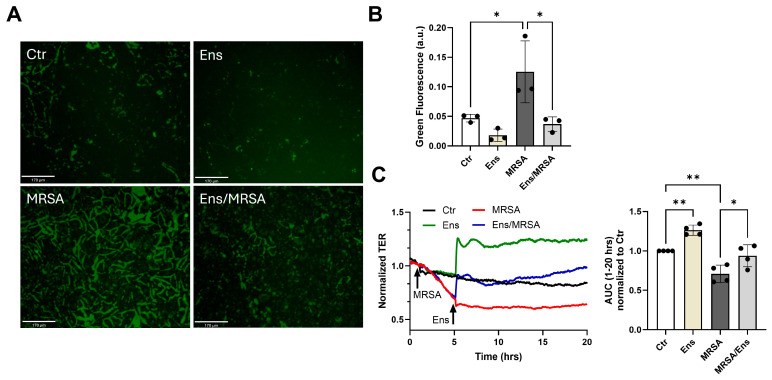
Ensifentrine inhibits MRSA-induced gap formation and restores barrier integrity. (**A**,**B**) XperT permeability assay. HPAEC grown on biotinylated gelatin-coated dishes were pre-treated with ensifentrine (5 μΜ) or vehicle (DMSO) for 1 h prior to HK-MRSA challenge (2.5 × 10^8^/mL). Then, 20 h later, FITC-avidin was added and pictures were taken at 20×. (**A**) Increased FITC signal indicates gap formation. Scale bar = 170 μm. (**B**) Quantification of green fluorescence using ImageJ. (**C**) HPAEC were treated with HK-MRSA (2.5 × 10^8^/mL), and 4 h later ensifentrine (5 μΜ) or vehicle (DMSO) was added. Depicted are representative TER tracings over time. The area under the curve was calculated for each condition for the period 1–20 h. N = 3–4 independent experiments. Data were analyzed using one-way ANOVA. * *p* < 0.05, ** *p* < 0.01.

To further explore the effects of dual PDE3/4 inhibition after MRSA challenge in lung EC, we employed the XperT assay, which allows the visualization of interendothelial gaps [26]. As expected, MRSA treatment caused gap formation, as indicated by the intense green signal in the representative images depicted and the corresponding quantification (Figure 2A,B). In cells pre-treated with ensifentrine, interendothelial gaps were dramatically reduced after MRSA, confirming its potent EC barrier-protective properties (Figure 2A,B). Next, we investigated whether ensifentrine could reverse the endothelial damage caused by MRSA. For this, HPAEC were treated with MRSA and ensifentrine was added 4 h later. As shown in the representative graph and the corresponding quantification (Figure 2C), ensifentrine reversed MRSA-induced EC permeability, even when added post-injury.

### 3.2. Mechanisms through Which Ensifentrine Protects against MRSA-Induced Lung EC Barrier Disruption

**Figure 3:**
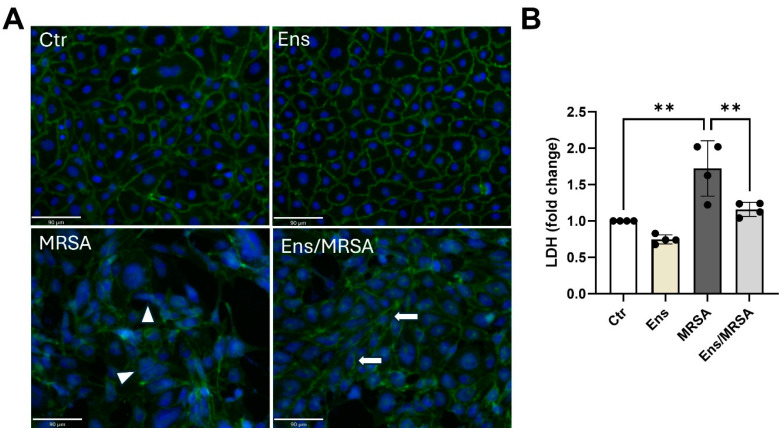
Ensifentrine attenuates MRSA-induced VE-cadherin disruption and cell death. HPAEC were pre-treated with ensifentrine (5 μM) or vehicle (DMSO) for 1 h prior to HK-MRSA challenge (2.5 × 10^8^/mL, 20 h). (**A**) EC were fixed and processed for VE-cadherin staining using an alexa-488 secondary antibody. Nuclei were stained using DAPI (blue). Images were taken at 20×. (Scale bar = 90 μm). Depicted are representative images. White arrows indicate VE-cadherin staining at cell junctions, and triangles indicate VE-cadherin bond disruption. (**B**) Extracellular LDH levels were measured in the conditioned media. N = 3–4 independent experiments. Data were analyzed using one-way ANOVA, ** *p* < 0.01.

The endothelial monolayer is maintained by intercellular junctions. Among these, adherens junctions are primary regulators of the lung endothelial barrier [29]. To determine whether ensifentrine impacts these junctions, HPAEC were processed for immunofluorescence and staining for VE-cadherin. As demonstrated in Figure 3A, VE-cadherin staining at cell–cell junctions is enhanced in ensifentrine-treated cells compared to control cells, providing additional evidence for the barrier-enhancement properties following PDE3/4 inhibition. MRSA caused a marked disruption of VE-cadherin junctions, which was attenuated in the presence of 5 μΜ ensifentrine (Figure 3A). Increased cell death is another mechanism by which the EC barrier integrity is compromised in ALI, leading to increased vascular permeability and impaired tissue function [30]. In lung EC treated with MRSA, there was a slight but significant increase in extracellular levels of LDH, which serves as a marker of cell death (Figure 3B). Importantly, in the lung EC pre-treated with ensifentrine, following MRSA, the LDH levels were significantly reduced to baseline levels (Figure 3B). Taken together, these data suggest that ensifentrine protects the EC barrier by mechanisms that involve both the maintenance of adherens junctions and a reduction in cell death.

### 3.3. Ensifentrine Exerts Anti-Inflammatory Effects in Lung EC Treated with MRSA

**Figure 4:**
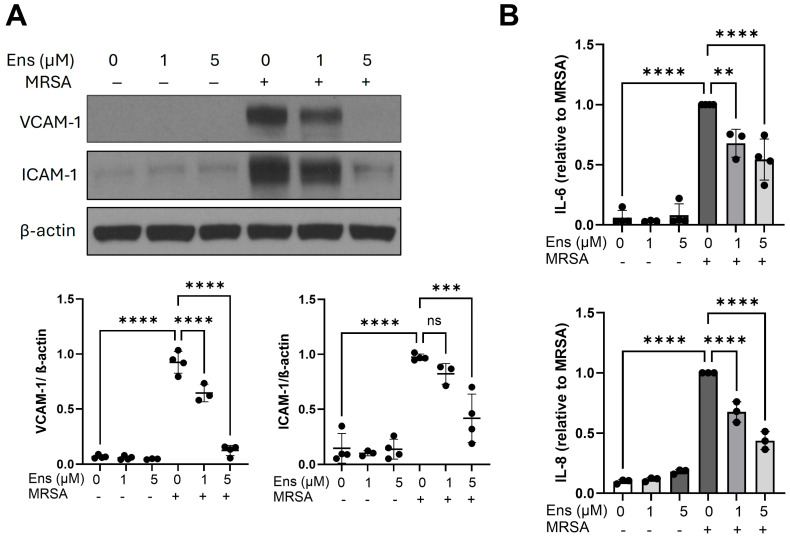
Ensifentrine inhibits MRSA-induced pro-inflammatory signaling in lung endothelial cells. HPAEC were pre-treated with ensifentrine (1 or 5 μM) or vehicle (DMSO) for 1 h prior to HK-MRSA challenge (2.5 × 10^8^/mL, 20 h). (**A**) Representative Western blots and pooled densitometric analyses of EC lysates are shown for VCAM-1, ICAM-1, and β-actin expression. (**B**) IL-6 and IL-8 levels were measured in EC supernatants. N = 3–4 independent experiments. Data were analyzed using one-way ANOVA, ns not significant, ** *p* < 0.01, *** *p* < 0.001, **** *p* < 0.0001.

PDE inhibitors are potent modulators of inflammation, so we investigated inflammatory pathways induced by MRSA in lung EC next. The expression of cell adhesion molecules, including vascular cell adhesion molecule 1 (VCAM-1) and intercellular adhesion molecule 1 (ICAM-1), is strongly upregulated in EC upon inflammatory stimulation to mediate leukocyte trafficking [31]. As shown in Figure 4A, MRSA caused a dramatic increase in the expression of both VCAM-1 and ICAM-1. However, ensifentrine, in a dose-dependent manner, significantly inhibited the induction of both proteins following MRSA (Figure 4A and Supplementary Figure S2B,C). At a concentration of 5 μM, ensifentrine suppressed VCAM-1 levels nearly to baseline and reduced ICAM-1 levels by 63% (Figure 4A). Moreover, MRSA-induced IL-6 and IL-8 levels were significantly decreased by 51% and 56%, respectively, in cells pre-treated with 5 μΜ of ensifentrine, as demonstrated in Figure 4B and Supplementary Table S1 (IL-6 and IL-8 raw values). The effects of 10 μΜ ensifentrine on IL-6 levels are shown in Supplementary Figure S2D.

### 3.4. Dual Inhibition of PDE3/4 in Alveolar Epithelial Cells Decreases Inflammation Following MRSA Exposure

**Figure 5:**
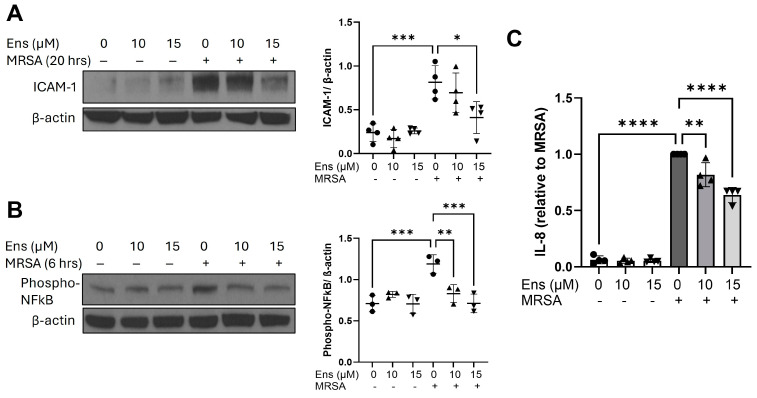
MRSA-induced inflammatory signaling in A549 is decreased by ensifentrine. A549 were pre-treated with ensifentrine (10 or 15 μM) or vehicle (DMSO) for 1 h prior to HK-MRSA challenge (2.5 × 10^8^/mL). Representative Western blots of cell lysates are shown for (**A**) ICAM-1 expression (20 h of MRSA) and (**B**) phospho-NFkB (6 h of MRSA). Densitometric analysis was performed by normalizing to β-actin levels. (**C**) IL-8 levels were measured in cell supernatants. N = 3–4 independent experiments. Data were analyzed using one-way ANOVA, * *p* < 0.05, ** *p* < 0.01, *** *p* < 0.001, **** *p* < 0.0001.

Alveolar epithelial cells (AEC) are in the first line of defense against respiratory pathogens, including MRSA, and along with the endothelium, they play a primary role in maintaining the integrity of the alveolar–capillary barrier. ARDS-relevant stimuli activate inflammatory signaling in AEC, which results in leukocyte recruitment and the propagation of inflammation. As shown in Figure 5A, MRSA caused a significant induction of ICAM-1, which was inhibited by ensifentrine in a dose-dependent manner. In addition, ensifentrine decreased the levels of phosphorylated NFkB induced by MRSA (Figure 5B) and reduced the release of the pro-inflammatory cytokine IL-8 (Figure 5C, Supplementary Table S1).

**Figure 6:**
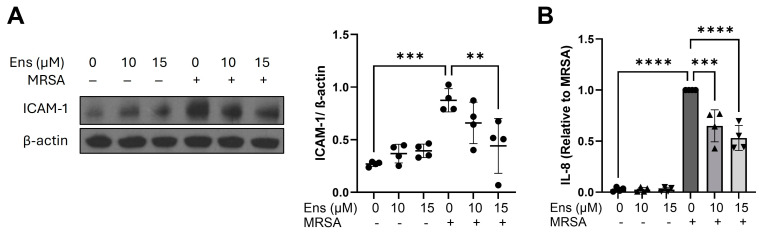
MRSA-induced inflammatory signaling in immortalized alveolar epithelial cells is decreased by ensifentrine. Immortalized AEC were pre-treated with ensifentrine (10 or 15 μM) or vehicle (DMSO) for 1 h prior to HK-MRSA challenge (3 × 10^8^/mL, 20 h). (**A**) Representative Western blots and pooled densitometric analyses of cell lysates are shown for ICAM-1 expression and β-actin. (**B**) IL-8 levels were measured in cell supernatants. N = 4 independent experiments. Data were analyzed using one-way ANOVA, ** *p* < 0.01, *** *p* < 0.001, **** *p* < 0.0001.

Complementary to A549, we also employed human immortalized AEC and assessed the effects of ensifentrine following MRSA treatment. Similar to A549, MRSA induces the upregulation of ICAM-1 expression and IL-8 release (Figure 6A,B). However, in cells pre-treated with 15 μΜ ensifentrine, both these inflammatory markers were significantly reduced by ~50% and ~47%, respectively (Figure 6A,B). The IL-8 raw values are shown in Supplementary Table S1. Note that in the epithelial cells (both A549 and iAEC), the IL-6 levels were very low at baseline and did not appreciably increase after MRSA treatment.

### 3.5. Epac Mediates Ensifentrine’s Protective Effects on Lung Endothelial and Epithelial Cells

**Figure 7:**
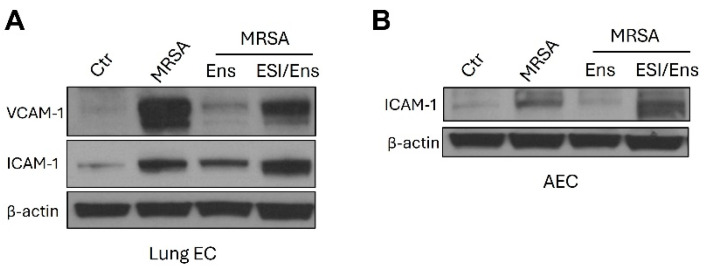
Ensifentrine reduces the expression of inflammatory adhesion molecules in lung EC and AEC in an EPAC-dependent manner. HPAEC and A549 were pre-treated with 20 μΜ or 5 μΜ ESI-08 (EPAC antagonist), respectively. After 1 h, ensifentrine was added (5 μM for HPAEC and 15 μΜ for A549). Cells were treated with HK-MRSA 1 h later (2.5 × 10^8^/mL, 20 h). Representative Western blots are shown for (**A**) VCAM-1 and ICAM-1 expression and β-actin in HPAEC, and (**B**) ICAM-1 expression and β-actin in A549. N = 3 independent experiments.

Previous studies have demonstrated that EPAC (exchange protein activated by cyclic AMP) is a critical target of PDE4 inhibitors [32], and its activation is essential for EC barrier maintenance and protection against inflammatory signaling [33,34]. To assess whether ensifentrine mediates its effects through EPAC, lung EC and A549 were pre-treated with the EPAC antagonist, ESI-08, prior to ensifentrine and MRSA treatments. As depicted in Figure 7A,B, in the presence of ESI-08, the effects of ensifentrine on the expression of adhesion molecules VCAM-1 and ICAM-1 were reduced in HPAEC, as well as its effects on ICAM-1 in A549 (densitometry is provided in Supplementary Figure S3). A lower dose (5 μΜ) of ESI-08 was used for A549 compared to HPAEC (20 μΜ) because doses 10 μM and higher caused significant cell death in these AEC. These data demonstrate that EPAC activation participates in the protective effects of ensifentrine in MRSA-treated lung cells.

## 4. Discussion

Our study characterized for the first time the effects of ensifentrine, a dual PDE 3/4 inhibitor, on lung endothelial and alveolar epithelial cell dysfunction caused by MRSA. MRSA is a Gram-positive pathogen that can cause severe pneumonia or sepsis-induced ARDS [5,35]. Here, we demonstrate that ensifentrine exerts endothelial barrier-protective properties pre- or post-MRSA treatment. Notably, the preservation of endothelial cell barrier integrity by ensifentrine was accompanied by suppressed inflammation in both lung EC and alveolar epithelial cells. Optimal lung health is dependent on a functional alveolar–capillary barrier, making strategies that protect both the lung endothelium and the epithelium from inflammatory insults advantageous and more promising.

There has been a sustained and long-term interest in phosphodiesterase 3 and 4 inhibitors and their therapeutic potential. Cilostazol and milrinone (PDE3i), along with roflumilast and apremilast (both PDE4i), are among the FDA-approved inhibitors developed for the treatment of various conditions [10]. Cilostazol is primarily used for peripheral arterial disease and milrinone is used for the short-term treatment of acute decompensated heart failure, while roflumilast and apremilast are FDA-approved for COPD and psoriasis, respectively [10]. These approved inhibitors, along with several other investigational selective PDE3 or 4 inhibitors, have been considered as potential therapeutics for additional indications, including the treatment of ARDS [14] and neurodegenerative diseases like Alzheimer’s and Parkinson’s [36].

Specifically for ALI pathophysiology, numerous pre-clinical studies have generated substantial evidence to demonstrate that the specific inhibition of PDE3 or 4 (as well as other PDE subtypes) suppresses lung inflammation, edema formation, lung epithelial and endothelial injury, and reduces platelet activation (reviewed in detail in [14]). Several clinical trials have also investigated the therapeutic potential of non-selective PDE inhibitors in ARDS (reviewed in [14]), with some of those showing promising results. However, there have been no clinical studies to test specific PDE4 inhibitors, and only two clinical studies have assessed the effects of PDE3i by milrinone in ARDS-related conditions [14]. The first study enrolled pediatric patients with non-hyperdynamic septic shock, and the second recruited severe sepsis patients [37,38]. Although data from both studies were positive, there has been limited interest in their widespread clinical use in ARDS, potentially due to their several adverse side effects, as discussed below.

Ensifentrine (RLP554) is a novel dual inhibitor of PDE3 and PDE4 that was recently (June 2024) approved by the FDA as a maintenance treatment for patients with COPD [18,39,40]. By targeting both PDE 3 and 4 enzymes, ensifentrine has been shown to relax the airway’s smooth muscles and suppress the release of pro-inflammatory mediators [18]. In addition, ensifentrine stimulates the cystic fibrosis transmembrane conductance regulator (CFTR) in *in vitro* studies, which can improve mucociliary clearance by reducing mucus viscosity and pathogens from the respiratory track [39,41]. In addition to COPD, ensifentrine is being considered for the treatment of asthma [42,43], cystic fibrosis, and non-cystic fibrosis bronchiectasis.

Despite its excellent therapeutic profile, there are only a few pre-clinical studies exploring the effects of ensifentrine on lung cell function. This present study aimed to determine the role of dual PDE3/4 inhibition by ensifentrine using an in vitro model of lung injury. Increased endothelial permeability is a critical step in the development and progression of ARDS [30]. Extensive research has demonstrated that phosphodiesterases and cyclic nucleotide second messengers (cAMP and cGMP) regulate endothelial barrier function [15]. cAMP and cGMP exhibit differential effects on endothelial permeability; however, the majority of evidence indicates that increased cAMP levels due to PDE inhibition results in the activation of PKA (protein kinase A) and EPAC signaling that mediate barrier enhancement and protection [15]. Indeed, several studies have demonstrated that the individual inhibition of PDE3 or 4, or their combination, leads to barrier protection. For example, the selective inhibition of PDE4 (by roflumilast) or PDE3 (by motapizone) reduced thrombin-induced macromolecule permeability in HUVEC, while their combination completely prevented it [17]. Early studies also demonstrated that an experimental PDE3/4 inhibitor, zardaverine, protected against thrombin and *Escherichia coli* hemolysin-induced lung endothelial barrier disruption [44]. In agreement with these prior observations, we present evidence that ensifentrine exhibits potent barrier-enhancing properties and barrier-protective effects against MRSA when given pre- or post-treatment (Figure 1 and Figure 2). These responses appear to be mediated by protection of the VE-cadherin junctions, which are cell–cell junctions that stabilize the endothelial monolayer but are disrupted by MRSA treatment, as we have previously shown [23]. Increased cell death, such as apoptosis and necroptosis, can compromise the integrity of the endothelial barrier [45,46]. MRSA causes a mild decrease in cellular viability as indicated by increased LDH levels, which was inhibited in the presence of ensifentrine (Figure 3B). It is therefore possible that the dual inhibition of PDE3/4 leads to barrier protection by preserving the interendothelial junctions and preventing cellular death.

The potent anti-inflammatory properties of PDEi are well-established [47,48]. In agreement with the literature, ensifentrine reduces pro-inflammatory signaling in lung EC in the current study. Specifically, this compound completely prevented VCAM-1 upregulation after MRSA and decreased the levels of ICAM-1 in endothelial cells (Figure 4). VCAM-1 and ICAM-1 are adhesion molecules, which are upregulated upon endothelial activation from inflammatory insults and mediate pro-inflammatory signaling and immune cell recruitment [49]. Strategies that reduce their expression mitigate ALI [50]. Prior research has shown that PDE3i by cilostazol downregulates VCAM-1 in the endothelium of diabetic rats [51], while PDE4i by apremilast reduces VCAM-1 but not ICAM-1 expression in TNF-α-treated HUVEC [52]. Combining a PDE3 inhibitor, Org9935, and the PDE4 inhibitor, rolipram, resulted in a synergistic reduction in VCAM-1 following TNF-α treatment in HUVEC. However, in the study that considered this combination, it did not affect ICAM-1 levels [53]. Our studies also demonstrated that IL-6 and IL-8 levels were significantly reduced following ensifentrine treatment in endothelial cells, consistent with the existing literature showing decreased inflammatory cytokine release following PDE3 or 4 inhibition [8,54].

In this study, MRSA caused the upregulation of ICAM-1, the activation of NF-kB, and the increased release of IL-8 in alveolar epithelial cells, all of which were reduced in the presence of ensifentrine (Figure 5 and Figure 6). In one of the few published studies exploring the role of ensifentrine in vitro, it was demonstrated that in well-differentiated bronchial epithelial cells that express a CFTR mutation (in vitro model of cystic fibrosis), ensifentrine reduced IL-1β-induced MCP1 and GM-CSF production, while it had no effect on IL-8 [21]. The anti-inflammatory properties of ensifentrine in this study were shown to be mediated by inhibition of PDE4, and this is consistent with the extensive literature studying the role of PDE4 and not PDE3 in epithelial cells, with a focus on bronchial epithelial cells. Less is known about PDE4 inhibition specifically in the alveolar epithelium, which is a primary site of ARDS pathogenesis. One study showed that PDE4 inhibition by roflumilast decreases the production of IL-8, MCP-1, and CXCL1 induced by neutrophil elastase in A549 cells [55]. Despite the positive effects of ensifentrine on AEC and EC, further research is needed to assess its impact in more physiologically relevant models, such as those using an air–liquid interface and co-culture systems.

While our studies clearly demonstrate that ensifentrine exerts potent barrier-protective and anti-inflammatory properties, its efficacy in vivo to treat preclinical models of ARDS remains to be determined. Important considerations for animal experiments are the dose and route of administration, which are especially important when using PDE inhibitors. It is well established that the systemic administration of PDEi is associated with several side effects that have limited their use, such as gastrointestinal issues [14]. Clinical trials testing inhaled ensifentrine for its safety and tolerability show that it is well-tolerated, with side effects comparable to those of a placebo [22]. In addition, our results demonstrate that endothelial cells require slightly lower doses of ensifentrine compared to epithelial cells. Therefore, the delivery of the medication by inhalation could potentially achieve higher concentrations in the alveoli compared to the vasculature and therefore efficiently target both cell types. Future studies will investigate ensifentrine’s efficacy in treating lung injury when given systemically versus directly in the respiratory system. Although the systemic route may not be optimal for patients given the side effect profile, preclinical studies using this route may help to determine the ensifentrine concentration required to achieve favorable results. In vivo experiments using live MRSA would also be necessary to evaluate the effects of ensifentrine, not only on edema and inflammation, but also on the immune response and bacteria clearance. Findings from preclinical studies on the efficacies of PDE 3 or 4 inhibitors in bacterial models show discrepancies. For example, although PDE4 inhibition by roflumilast impairs *Pseudomonas aeruginosa* in the airways [56], PDE3 inhibition by cilostazol disrupts *P. aeruginosa* growth and infection [57]. In a pneumococcal pneumonia model, rolipram (PDE4i) reduced lung injury levels, without affecting the bacterial burden [58]. Interestingly, a recent study demonstrated in a model of MRSA-induced bacteremia that the administration of roflumilast-containing nanoparticles specifically targeting the neutrophils alleviated MRSA infection and organ injury [59]. Understanding the impact of PDE3/4 inhibition on the immune system is important since there have been reports suggesting that COPD patients on PDE4i may be more susceptible to pneumonia [60]. Conversely, other studies propose that PDE3i in patients with other comorbidities is associated with a reduced incidence of pneumonia [61]. To date, clinical studies testing ensifentrine have not shown an association between its use and an increased or reduced risk of non-COVID-19 pneumonia [18]. However, new studies are needed, not only to comprehensively assess the effects of ensifentrine on immune cell functions and host–pathogen interactions in the lung, but also to evaluate the efficacy of ensifentrine towards other ARDS-related pathogens, including *Streptococcus pneumoniae* and *P.aeruginosa*.

Further research is also required to explore the mechanisms by which ensifentrine mediates its potent effects. EPAC and PKA are directly activated by cAMP, and they are downstream effectors of PDEi [15]. A recent study demonstrated that EPAC activation by 8CPT in lung endothelial cells stimulated with extracellular histones led to superior barrier-enhancing and protective properties, and it caused dramatic decreases in several inflammatory markers including VCAM-1, ICAM-1, and pro-inflammatory cytokines [34]. In agreement with this, our data (Figure 7) suggest that EPAC activation at least partially mediates ensifentrine’s protective effects on both lung EC and AEC. However, additional studies are needed to elucidate whether PKA or other cAMP-independent signaling pathways are involved. Finally, since ensifentrine exhibits varying selectivity for PDE3 versus PDE4, and the relative expression of PDE3 and PDE4 differs depending on the cell type, it remains to be determined which PDE isoenzyme is most responsible for each effect.

## 5. Conclusions

In summary, our study demonstrates that the dual inhibition of PDE 3 and 4 by the newly FDA-approved compound, ensifentrine, protects against MRSA-induced lung endothelial and alveolar epithelial dysfunction. As the disruption of the alveolar–capillary permeability is a primary step in the pathogenesis and progression of ARDS, our data suggest that ensifentrine is a promising strategy for maintaining lung barrier integrity and suppressing inflammation in ALI. Ensifentrine may represent a new therapeutic option for managing MRSA-induced ARDS and other inflammatory diseases, and more research is needed to fully understand its potential.

## Data Availability

Data are available on reasonable request.

## Supplementary Materials

The following supporting information can be downloaded at https://www.mdpi.com/article/10.3390/cells13211750/s1: Figure S1: Early effects of ensifentrine on EC barrier function; Figure S2: Effects of ensifentrine (10 μΜ) on lung EC barrier function after MRSA treatment; Figure S3: EPAC antagonism by ESI-08 alters the effects of ensifentrine on adhesion molecule expression in lung EC and AEC exposed to MRSA; Supplementary Table S1: IL-6 and IL-8 raw values.
