# Supplementary material for: Dual Inhibition of Phosphodiesterase 3 and 4 Enzymes by Ensifentrine Protects against MRSA-Induced Lung Endothelial and Epithelial Dysfunction
Source: Cells. 2024 Oct 23;13(21):1750. doi: 10.3390/cells13211750 (PMC11545647; doi:10.3390/cells13211750)
Supplement: Supplementary file 1 [file cells-13-01750-s001.zip › Supplementary/Supplementary figs.revised-1021.pdf]

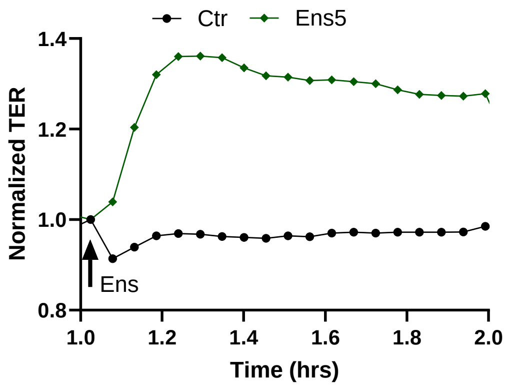

Supplementary Figure 1. Early phase effects of ensifentrine on EC barrier function. Human pulmonary artery endothelial cells (HPAEC) were treated with ensifentrine (5 μM) or vehicle (DMSO). EC barrier function was assessed using the ECIS assay. Depicted is a representative plot of normalized TER values recorded for 1 hour after addition of ensifentrine.

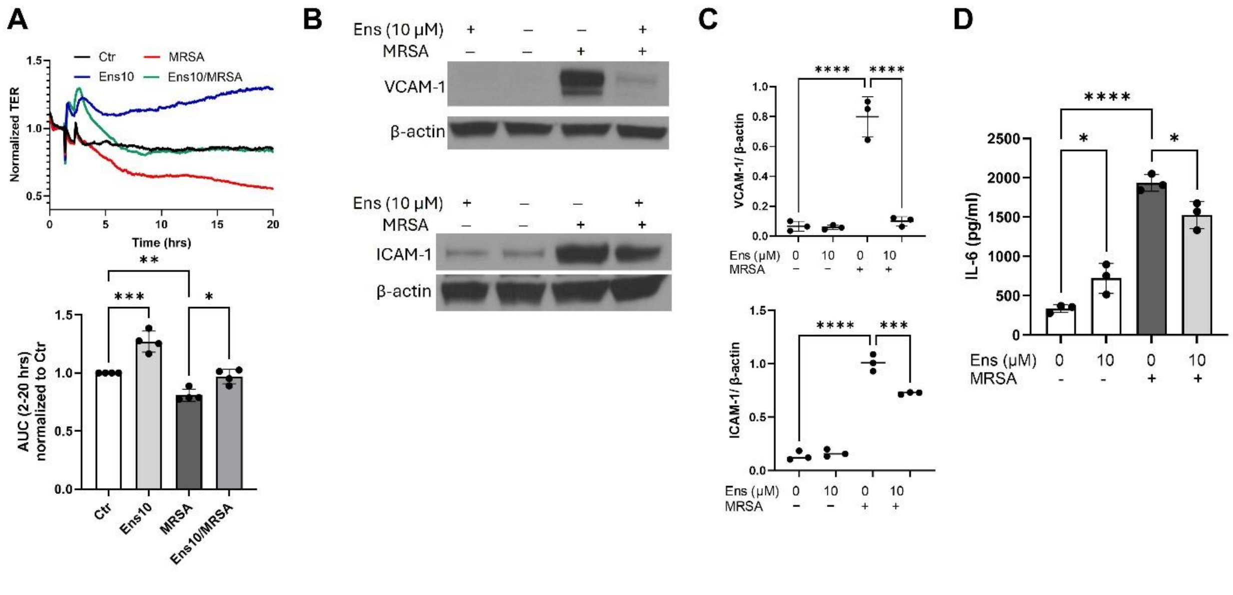

Supplementary Figure 2. Effects of ensifentrine (10 μM) on lung EC barrier function after MRSA treatment. HPAEC were pre-treated with ensifentrine (10 μM) or vehicle (DMSO) for 1 hour prior to HK-MRSA challenge (2.5 x 10^8/ml). EC barrier was assessed with the ECIS assay. (**A**) Representative TER tracings over time. Data were quantified after calculating the area under the curve for each condition. (**B-C**) Representative western blots of EC lysates for VCAM-1 and ICAM-1 expression and corresponding densitometry. (**D**) IL-6 levels were measured in EC

supernatants. N=3-4 independent experiments. Data were analyzed using one-way Anova, *p<0.05, **p<0.01, ***p<0.001, ****p<0.0001.

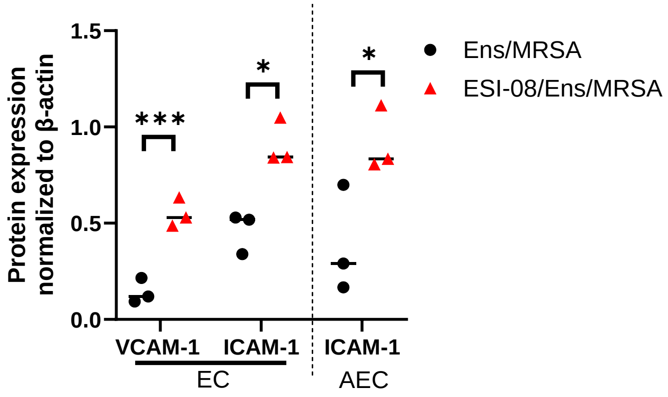

Supplementary Figure 3. EPAC antagonism by ESI-08 alters the effects of ensifentrine on adhesion molecule expression in lung EC and AEC exposed to MRSA. HPAEC and A549 were pre-treated with 20 µM or 5 µM ESI-08 (EPAC antagonist) respectively. After 1 hour, ensifentrine was added (5 µM for HPAEC and 15 µM for A549). Cells were treated with HK-MRSA 1 hour later (2.5 $x10^8$/ml, 20 hrs), and cell lysates were analyzed for VCAM-1 and ICAM-1 expression. Depicted is the densitometry analysis. Data were analyzed using t-test, *p<0.05, ***p<0.001.
