# Supplementary material for: Dual Inhibition of Phosphodiesterase 3 and 4 Enzymes by Ensifentrine Protects against MRSA-Induced Lung Endothelial and Epithelial Dysfunction
Source: Cells. 2024 Oct 23;13(21):1750. doi: 10.3390/cells13211750 (PMC11545647; doi:10.3390/cells13211750)
Supplement: Supplementary file 1 [file cells-13-01750-s001.zip › Supplementary/supplementary table 1.pdf]

Supplementary Table 1. IL-6 and IL-8 raw values.

| **HPAEC** | | | | **IL-6 (pg/ml)** | | |
|---|---|---|---|---|---|---|
| | **Ctr** | **Ens 1 μM** | **Ens 5 μM** | **MRSA** | **Ens 1 μM/MRSA** | **Ens 5 μM/MRSA** |
| | 81.722 | 80.586 | 87.996 | 2830.772 | 2089.296 | 1504.568 |
| | 63.062 | 73.012 | 84.294 | 1940.612 | 1058.664 | 639.06 |
| | 68.22 | 70.984 | 73.796 | 2163.428 | 1631.208 | 1227.404 |
| | 62.706 | | 91.966 | 416.008 | | 310.716 |

| **HPAEC** | | | | **IL-8 (pg/ml)** | | |
|---|---|---|---|---|---|---|
| | **Ctr** | **Ens 1 μM** | **Ens 5 μM** | **MRSA** | **Ens 1 μM/MRSA** | **Ens 5 μM/MRSA** |
| | 459.22 | 527.274 | 835.824 | 4328.132 | 3286.94 | 2225.44 |
| | 468.552 | 584.228 | 961.248 | 6040.588 | 3559.376 | 2205.04 |
| | 534.522 | 619.508 | 921.502 | 4876.84 | 3312.036 | 2127.164 |

| **A549** | | | | **IL-8 (pg/ml)** | | |
|---|---|---|---|---|---|---|
| | **Ctr** | **Ens 10 μM** | **Ens 15 μM** | **MRSA** | **Ens 10 μM/MRSA** | **Ens 15 μM/MRSA** |
| | 42.1 | 41.3 | 35.9 | 735.3 | 530.6 | 492.9 |
| | 27.7 | 34.4 | 47.7 | 1111.5 | 1076.7 | 748.8 |
| | 45.3 | 35.1 | 33.2 | 414.4 | 336.8 | 278.3 |
| | 51.7 | 41.7 | 43.6 | 955.9 | 737.0 | 514.0 |

| **iAEC** | | | | **IL-8 (pg/ml)** | | |
|---|---|---|---|---|---|---|
| | **Ctr** | **Ens 10 μM** | **Ens 15 μM** | **MRSA** | **Ens 10 μM/MRSA** | **Ens 15 μM/MRSA** |
| | 78.768 | 76.561 | 62.102 | 1523.91 | 654.756 | 663.879 |
| | 7.49 | 3.5 | 3.5 | 503.416 | 324.215 | 277.869 |
| | 7.296 | 9.644 | 9.09 | 1288.719 | 989.694 | 895.671 |
| | 48.243 | 49.702 | 67.449 | 1353.249 | 1025.466 | 597.204 |
